# Health Effects of Energy Intensive Sectors and the Potential Health Co-Benefits of a Low Carbon Industrial Transition in China

**DOI:** 10.3390/ijerph16173022

**Published:** 2019-08-21

**Authors:** Tingru Yang, Wenling Liu

**Affiliations:** 1 Center for Energy and Environmental Policy Research, Beijing Institute of Technology, Beijing 100081, China; 2 School of Management and Economics, Beijing Institute of Technology, Beijing 100081, China; 3 Sustainable Development Research Institute for Economy and Society of Beijing, Beijing 100081, China; 4 Beijing Key Lab of Energy Economics and Environmental Management, Beijing 100081, China

**Keywords:** health effect, health economic loss, co-benefit, exposure-response relationship, energy intensive sectors

## Abstract

*Background:* The issues of environmental pollution and its effects on health have become increasingly serious in China. Energy intensive sectors are not only the main energy consumers, but also the main sources of air pollution. Analyzing the health effects of energy intensive sectors and the potential health co-benefits of a low carbon industrial transition is of great importance for promoting China’s air pollution control. *Methods:* This study used the exposure-response (ER) relationship model and inhalation factor methods to quantitatively analyze the health effects of air pollution and forecast the potential health co-benefits in the power and steel sectors. *Results:* The results showed that in 2016 SO_2_ and PM_2.5_ emissions caused about 850,000 premature deaths, and 10 million cases of respiratory diseases and chest discomfort, resulting in health-related economic losses of 1.2 trillion Yuan, accounting for 1.6% of the GDP. Meanwhile, demand control in consumption could significantly reduce SO_2_ emissions in the power and steel sectors, thus offering significant health co-benefits. However, there was still some uncertainty regarding the reduction of PM_2.5_ emissions in the steel sector. *Conclusions:* There is a need to take advantage of the health co-benefits of emission reduction in energy intensive sectors and to adopt flexible means to stimulate their green transformation.

## 1. Introduction

In parallel with the rapid development of the economy and the acceleration of urbanisation and industrialisation, the issue of environmental pollution has become increasingly serious in China. Severe environmental pollution endangers public health, causes medical burdens and even jeopardises energy security and social stability. It has become a huge challenge for the development of China. As shown by the Chinese Ecological Environment Status Bulletin 2017, among the 338 prefecture-level cities in China, 239 failed to meet air quality standards and the proportion of polluted cities reached 22%, of which 2.6% were heavily polluted (The degree of air pollution is reflected by Air Quality Index (AQI). “Polluted city” means the AQI is greater than 100; "heavily polluted" means the AQI is greater than 300.) [1]. The air pollution situation in China is very serious. Although air quality has improved slightly in recent years, there is still a long way to go to achieve healthy air quality standards.

People live in an environmental system and are inevitably exposed to environmental pollution caused by exhaust gases and solid emissions [2]. Since the 1980s, the health effects of environmental pollution have gradually been confirmed. Many studies have used the exposure-response (ER) relationship model [3,4,5] to explore the effect of air pollution on mortality [6,7,8], life expectancy, cardiopulmonary [9], cardiovascular and respiratory diseases [10,11], mental health, hospitalisation rates, and so on. Numerous quantitative studies have shown that China’s main air pollutants have caused damage to human health, as evidenced by increased mortality, respiratory diseases and hospitalisation rates. Air pollution not only directly affects human health, but also indirectly influences human production activities. Therefore, in addition to affecting the health of residents, the economic losses caused by air pollution are considerable.

To alleviate air pollution and its health effects in China, it is extremely important to find the sources of pollution and evaluate their effects on health. Since the policy of reforming and opening up in 1978, China’s economic development has produced remarkable results, with its rapid growth depending on the mass input of resource elements. Industrial GDP, which accounts for about 40% of the total GDP, consumes nearly 70% of energy. A International Energy Agency report [12] showed that the main contributors to air pollution globally are transportation, industry and power generation. According to China Energy Report 2018, “energy-intensive sectors” are those that consume and use large amounts of energy in the production process. In China, energy-intensive sectors mainly include steel, nonferrous metals, building materials, chemicals, electricity, and transportation sectors. Regarding the emissions of industrial pollutants in China, as shown by the Annual Report on Environmental Statistics in China 2015, among the 41 industrial sectors, the top three sectors for SO_2_ and NO_x_ emissions are the power, thermal production and supply industry, the non-metallic mineral products industry, and the ferrous metal smelting and rolling processing industry, all of which are also energy intensive sectors. SO_2_ emissions from these three sectors accounted for more than 60% of the total emissions of industrial enterprises, NO_x_ emissions for more than 75% and soot (dust) emissions for more than 70% [13]. In other words, energy intensive sectors are not only the main energy consumers, but also the main sources of air pollutants. Therefore, focusing on energy intensive sectors to examine the health effects of pollution is of great importance for promoting the reduction of national greenhouse gas emissions, controlling air pollution and improving public health. In addition, China has made great efforts to conserve energy and reduce emissions, especially in energy intensive sectors, inevitably bringing additional costs and burdens to industries and enterprises. Nevertheless, emission reduction may have certain health co-benefits. Assessing these co-benefits can help provide a reference for the design of energy conservation and emission reduction policies, such as subsidy policies and cost-sharing mechanisms.

Based on the discussion above, this study focuses on the effects of pollutant emissions from energy intensive sectors in China, using the ER relationship model and inhalation factor methods to quantitatively analyse the health effects of air pollution. With scenario analysis, we then simulate the potential health co-benefits of energy conservation and emission mitigation in energy intensive sectors, taking the power generation and steel and iron sectors as examples.

The rest of this paper is organised as follows. Section 2 reviews the literature on the health effects of environmental pollution. Section 3 introduces the methods and data used in this study. Section 4 presents the results regarding the health effects of pollution in energy intensive sectors and the health co-benefits of energy conservation and emission reduction. Finally, Section 5 concludes the study.

## 2. Literature Review

People around the world are paying more and more attention to the reduction of environmental pollution, and air pollution and its related effects on public health have become an important issue in academia in recent years. Following Grossman [14], scholars have started to explore the effects of environmental pollution on public health. Early studies understood health as an economic capital, suggesting that the health effects of environmental pollution to a large extent depend on the likelihood of exposure to pollution [15]. As other conditions are set, the higher the level of exposure to environmental pollution, the greater the health risks and health hazards. In terms of research methodologies, early studies on the assessment of the health effects of environmental pollution mainly used the market value method, the opportunity cost method, the engineering cost method, the cost of illness approach, the traditional human resource method and the amended human resource method [16,17]. In recent years, scholars have mainly used the ER relationship method to evaluate environmental health effects. Most studies have used the ER relationship method with econometric models, such as Ordinary Least Square (OLS) and Generalized Additive Models (GAM), to assess these environmental health effects by estimating the effects of atmospheric pollutants on mortality rates [6,7,8], life expectancy [18,19], respiratory and cardiopulmonary diseases [10,11] and hospitalisation rates.

The health effects of environmental pollution have been further quantified by estimating the economic losses caused by the health effects of pollution [20,21,22]. Previous studies have made significant progress in the endogenous treatment of pollution-related health effects, pollution-related health damage and labour supply or productivity and pollution-related health damage and economic growth [23]. In particular, many studies have focused on pollution and health issues in China [24,25,26,27,28]. Numerous quantitative research results have shown that several major air pollutants in China cause damage to human health. In a study conducted in Beijing [29], the positive correlation between the atmospheric concentration of CO, SO_2_ and NO_X_ and the mortality rate of various diseases was significant, as the total suspended particulates increased to 10 μg/m^3^, the mortality rate from respiratory diseases increased by 3.19% and the mortality rate from diseases of the circulatory system increased by 0.62%. The studies by Chen et al. and Ebenstain et al. [18,26] were concerned with the health effects of China’s heating policies, showing that the average life expectancy of residents on the north side of the Huai River has decreased due to these heating policies. As Zhang et al. [30] showed, mental health is also influenced by pollution, with the deterioration of air quality affecting people’s psychological state, resulting in a decrease in the subjective well-being of individuals. Moreover, studies have shown that air pollution directly affects the health of residents and indirectly affects labour productivity, as air pollution leads to diseases that bring new economic burdens. Taking as an example the air pollutant PM_10_ in 2006, the analysis results from 113 cities in China showed that air pollutants caused huge health and economic losses to the residents of these Chinese cities, causing about 297,700 premature deaths and economic losses reaching 341.403 billion Yuan [31].

Concerns about the health effects of pollution are not always negative. In the process of tackling climate change, the mitigation goal has failed to mobilise sufficient mitigation efforts, which has led to more and more research on the co-benefits of greenhouse gas emission reduction. The co-benefits of greenhouse gas emission reduction with respect to their effects on ecosystems, economic activities, health, air pollution and resource efficiency have been the focus of current research [32]. Among them, health as a co-benefit of mitigation reveals the positive effects of climate policies in the short term and may compensate to some extent the costs of mitigation actions [33,34]. Most scholars have quantified the health co-benefits of the effects of climate change mitigation on air quality, transportation and diet. Scenario analysis has been widely used, with scenario settings including specific policy recommendations and hypothetical scenarios [35]. In measuring the health co-benefits of emission reduction in energy intensive sectors, many studies have measured these co-benefits in terms of mortality, morbidity, disease and health costs. This estimation method has been widely used in epidemiological research. It links public health effects, such as the reduction of mortality and healthcare costs, with the degree of human exposure to air pollutants. Some studies have shown that even a small increase in air pollutants would lead to increased health costs. For example, the study of Balbus et al. [36] on the transportation, construction and power sector in the US revealed that reducing PM_2.5_ emissions by 2020 would reduce healthcare costs from US $6 billion to US $3 billion. Other studies had similar results. For instance, Crawford-Brown et al. [37] showed that by 2020, Mexico’s mitigation policy would result in a reduction of 3000 deaths and 417,000 non-fatal diseases per year. Bailis et al. [38] focused on the health co-benefits of the household energy conversion process in Africa and estimated that converting household energy consumption from wood to charcoal and oil by 2030 would result in 1 to 1.3 million fewer deaths. Other studies have focused on the health co-benefits of non-CO_2_ greenhouse gas emission reduction. For example, Anenberg et al. [39] used the ER model to assess the health co-benefits of global CH_4_ and BC reduction policies.

In summary, most studies on the health co-benefits of emission reduction in energy intensive sectors have shown that short-term health co-benefits promote the implementation of mitigation policies and reduce net costs. However, these results have mainly described the interaction between climate policies and health and their potential effects, lacking an accurate estimation of health co-benefits. Many domestic and foreign studies have proved that environmental pollution, especially air pollution, can cause damage to health and lead to economic losses. They have built a pollutant-health response relationship, laying the foundation for understanding the health effects of pollution. However, most current research has assessed the health effects of pollutants at the gross level, while the health losses caused by different industries differ significantly. In addition, the assessment of the health effects of the main sources of pollution remains relatively rare. Energy intensive sectors are also important sources of air pollutants. Therefore, this study focused on the evaluation of the health effects and economic effects of energy intensive sectors to predict the potential health co-benefits of emission reduction and provide an important basis and decision-making reference for the corresponding cost and benefit analysis of various policies.

## 3. Data and Methodology

This study mainly used the ER relationship model to assess the health effects and economic losses of pollution in energy intensive sectors. Specifically, the following steps were required. First, it was necessary to estimate pollutant emissions from these energy intensive sectors and, using the inhalation factor method, to establish the relationship between pollutant emissions and public exposure to pollution. Then, using the ER relationship, health outcomes were linked with public exposure to pollution. By combining these two steps, the health effects and economic losses caused by each energy intensive sector were obtained. Subsequently, this study evaluated the health co-benefits of carbon mitigation in the power sector and steel sector through scenario analysis.

### 3.1. Methods for Assessing the Health Effects of Pollution and Their Economic Losses

This study assessed the environmental health effects and economic losses of air pollution. According to the results of the literature review, previous studies have mainly used the ER relationship method to evaluate environmental health effects. Therefore, this study used the revised ER relationship of Ho and Jorgenson [25] presented in Equation (1):

(1) $${HE}_{xrh}={ER}_{xh}\times C_{rx}\times {POP}_{r},$$

where ${HE}_{xrh}$ is the environmental health effects of h type of pollutant x in r area, ${ER}_{xh}$ is the ER relationship coefficient of h type of pollutant x, $C_{rx}$ is the concentration of pollutant x in r area and ${POP}_{r}$ is the population in r area.

The economic losses of environmental health effects were obtained by summing the measures of each health economic loss. The methods used are given by Equations (2) and (3):

(2) $${HEV}_{rxh}=V_{xh}\times HE_{xrh}$$

(3) $$\mathrm{THEV}=\sum_{r}\sum_{x}\sum_{h}HEV_{rxh}$$

where $HEV_{rxh}$ is the economic losses of h type of pollutant x in r area, $V_{xh}$ is the unit loss value of health effects by h type of pollutant x and THEV is the total economic losses caused by air pollution.

Regarding the measurement of ER relationship coefficients, Hammitt and Zhou [24], Ho and Jorgenson [25] and Gao et al. [40] used field research data to estimate the economic losses of various environmental health effects in China using the willingness to pay method. As the field research data were obtained in 2002, this study revised the coefficients with the 2016 price index based on Ho and Jorgenson’s [25] research data. The revised coefficients are shown in Table 1.

### 3.2. Calculation of Industrial Emissions and Public Exposure to Pollution

Following Wei et al. [41], the calculation of sectoral pollutant emissions in this study included fuel combustion emissions and industrial process emissions, as shown in Equations (4) to (6):

(4) $$EM_{jx}=EM_{jx}^{FC}+EM_{jx}^{P}$$

(5) $$EM_{jx}^{P}=\delta_{jx}OP_{j}$$

(6) $$EM_{jx}^{FC}=\sum_{f}\left(\psi_{jx}F_{j}\right)$$

where $EM_{jx}$ is the emissions of pollutant *x* in sector *j* and $EM_{jx}^{P}$ is the industrial process emissions, obtained using the emission rate $\delta_{jx}$ in the combustion process and output of sector j $OP_{j}$. $EM_{jx}^{FC}$ is the emissions from fuel combustion, estimated by emission factors $\psi_{jx}$ and fuel consumption $F_{j}$. The energy consumption data came from the China Energy Statistical Yearbook 2017 [42], the input-output data came from the Input-output Tables of China 2012 and the data on pollutant emissions and emission factors came from the GAINS model of the International Institute for Applied Systems Analysis (IIASA). After computing pollutant emissions from energy intensive sectors, it was necessary to establish the relationship between pollutant emissions and public exposure to pollution. The inhalation factor (iF, also known as exposure efficiency or exposure factor) can describe the quantitative relationship between pollutant emissions and the amount of pollutants inhaled by an exposed population. For a particular source of pollution, the corresponding inhalation factor can be expressed by Equation (7) [25]:

(7) $$\mathrm{iF}=\left(\sum_{r=1}^{n}POP_{r}\times C_{r}\times BR\right)/EM$$

where $POP_{r}$ is the population in area *r*, $C_{r}$ is the exposure concentration in area *r*, *BR* is the average respiratory rate of the exposed population, usually expressed as 20 m^3^/day and *EM* is the total emissions from specific sources of pollution.

Compared with other methods of assessing environmental health effects, the inhalation factor is a relatively new assessment tool, but has been widely used in assessing the environmental effects of power plants [43,44,45], industrial pollution [44], indoor pollution [46,47], traffic emissions [48] and some regional assessments [49]. Early studies [42,50,51] on the measurement of inhalation factors in urban China have mainly used the 1999 population data. Since 2000, urbanisation in China has accelerated and the urban population has increased rapidly. In 2016, China’s urbanisation rate reached 57.4%, while in 1999 it was only 34.8%, representing an increase of about 20%. A higher urbanisation rate means that more people are exposed to urban air pollution. Therefore, this study used the change in urbanisation rate to correct the original inhalation factor data, as shown in Table 2.

The environmental health effects of sectoral emissions were obtained using the ER relationship method. Sectoral pollutant emissions were then linked with pollutant intake using inhalation factors, as shown in Equation (8):

(8) $$INTAKE_{xj}=iF_{xj}^{N}\times EM_{jx}=BR\underset{r}{\sum }C_{xrj}POP_{r}$$

where $INTAKE_{xj}$ is the total public intake of pollutant *x* in sector *j*, $iF_{xj}^{N}$ is the inhalation factor of pollutant *x* in sector *j* and $C_{xrj}$ is the concentration of pollutant *x* in sector *j* in area *r*. Furthermore, the environmental health effects of pollutant *x* can be obtained by Equation (9):

(9) $$HE_{xhj}=ER_{hx}\times C_{x}\times POP$$

where $HE_{h}$ is the environmental health effects of *h* type of air pollutant *x*, $ER_{xh}$ is the ER relationship coefficient of *h* type of air pollutant *x*, $C_{x}$ is the average concentration of pollutant *x* in China and $POP_{r}$ is the exposed population. The environmental health effects of *h* type of air pollutant *x* in sector *j* is expressed by Equation (10):

(10) $$HE_{xhj}=\sum_{x}\left(ER_{hx}\times \frac{INTAKE_{xj}}{BR}\right)=\sum_{x}\left(ER_{hx}\times \frac{iF_{xj}^{N}\times EM_{xj}}{BR}\right)$$

### 3.3. Scenario Construction to Simulate Health Co-Benefits

To measure the health co-benefits of energy intensive sectors, this study used the power sector and the steel sector as examples to construct transition scenarios and predict their pollutant emissions under different scenarios. Health effects (and major economic losses) were estimated using the methods presented in the two subsections above.

The demand and emissions scenarios of the power and steel sectors were calculated by the Center for Energy and Environmental Policy Research of the Beijing Institute of Technology. For the power sector, following Tang et al. [52], this study designed five possible development strategies for power firms by investigating the effect of promoting advanced technologies and developing renewable energy technologies, including the business as usual (BAU) scenario, medium power demand (MPD) policy scenario, medium power demand (MPD) integrated scenario, power demand control (PDC) policy scenario and power demand control (PDC) integrated scenario. The definitions of the five scenarios are given in Table 3.

For the steel sector, following An et al. [56], this study considered six scenarios for SO_2_ and PM_2.5_ emissions, including medium, low and high demand for steel in the baseline and enhanced scenarios, using medium demand as the baseline scenario. The definitions of the six scenarios are given in Table 4.

## 4. Results

### 4.1. Health Effects of Pollutant Emissions in Energy Intensive Sectors

As shown in Table 5, in 2016, SO_2_ emissions in China’s main energy intensive sectors caused about 46,000 premature deaths each year, 23.42 million cases of chest discomfort and 5.85 million cases of lower respiratory tract infection and childhood asthma. In addition, PM_2.5_ emissions in these sectors caused approximately 800,000 non-accidental deaths, 90,000 deaths from respiratory diseases and 30,000 deaths from circulatory diseases. With only two contaminants considered, the number of premature deaths caused by energy intensive sectors was close to 1 million. As major sources of energy consumption and pollutant emissions, emissions from these sectors have huge negative effects on public health.

Based on the evaluation of health effects, this study also measured economic losses, as shown in Table 6. In China’s energy intensive sectors, the total economic losses caused by the health effects of SO_2_ emissions were about 273.94 billion Yuan, accounting for 0.37% of the national GDP that year. The total economic losses of PM_2.5_ emissions were about 929.1 billion Yuan, accounting for 1.25% of national GDP. When compared, PM_2.5_ emissions had greater health effects and health economic losses.

In contrast to current research results, many studies have confirmed that air pollution has already led to serious health problems and huge social costs for residents. For instance, Lelieveld et al. [59] calculated that PM_2.5_ emissions were responsible for 3.3 million premature deaths per year worldwide, predominantly in Asia. Similarly, Burnett et al. [60] concluded that PM_2.5_ emissions resulted in 8.9 million deaths worldwide in 2015, according to their latest calculations. In terms of health economic losses, the calculation of Barwick et al. [61] showed that if the PM_2.5_ concentration decreased by 10 μg/m^3^, it would save 9.2 billion Yuan in health expenditure, accounting for 1.5% of national health expenditure in China. These calculations focused on the total number of deaths and overall social welfare. However, this study only focused on energy intensive sectors, which caused about 800,000 premature deaths and led to health-related economic losses representing 1.6% of GDP.

**Figure 1:**
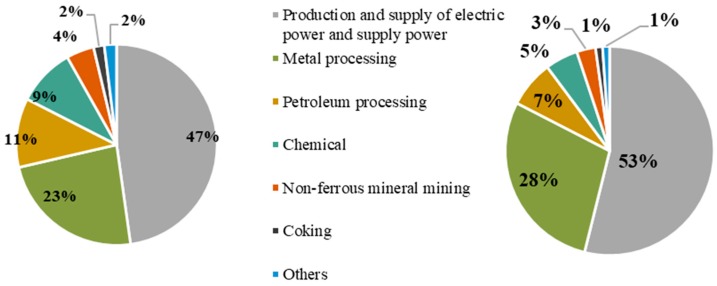
Contribution of different sectors to economic losses.

With regard to the contribution of different sectors, as shown in Figure 1, SO_2_ emissions from the production and supply of the electric power and supply power sector caused the largest economic losses, reaching 47% of the total economic losses (contribution of energy intensive sectors), followed by the metal processing sector, accounting for 23% of the total economic losses. The economic losses of the petroleum refining and chemical sectors were about 10%. Therefore, the health economic losses of these four sectors accounted for more than 90% of all energy intensive sectors. The situation of PM_2.5_ emissions was basically the same as before. The production and supply of the electric power and supply power sector caused the largest economic losses (53%), followed by the metal processing sector (28%), the petroleum refining sector (7%) and the chemical sector (5%). In general, the production and supply of the electric power and supply power sector and the metal processing sector contributed to significant economic losses. Compared with SO_2_ emissions, the economic losses of PM_2.5_ emissions in these two sectors were larger.

### 4.2. Health Co-Benefits of Carbon Mitigation in Energy Intensive Sectors

In recent years, along with increasing global responses to climate change, China has made great efforts to reduce greenhouse gas emissions, particularly for energy conservation and emission reduction in energy intensive sectors, with significant results. While mitigating greenhouse gas emissions from these energy intensive sectors, China has revealed certain co-benefits of reducing pollutant emissions and their negative health effects. For example, according to Watts et al.’s [2] study on the health co-benefits of energy saving and emission reduction technologies, low-carbon transitions in the areas of energy, transportation and agriculture enable residents to increase their exercise (for instance, through greater involvement in green transportation) and reduce the risk of diseases caused by environmental pollution. The co-benefits of greenhouse gas emissions reduction have positive effects on ecosystems, economic activities, health, air pollution and resource efficiency. Among them, health co-benefits reveal the positive effects of climate policies in the short term and can offset to a certain extent the costs of mitigation actions. At the same time, health co-benefits are closer to the public interest and can generate benefits faster, thus facilitating the implementation of climate policies.

According to the assessment of the health effects of energy intensive sectors in the previous section, the health effects and related economic losses of the power and metal processing sectors accounted for nearly 80% of all energy intensive sectors. Therefore, this study used the power sector and the steel sector as examples to construct transition scenarios and predict pollutant emissions from different scenarios. For more details on the transition scenarios, please see Tang et al. [52] and An et al. [56].

**Figure 2:**
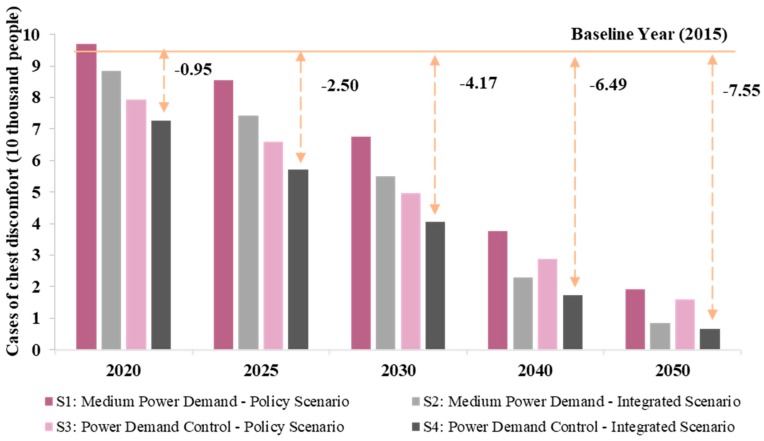
Health effects of SO_2_ emissions under different scenarios in the power sector.

Due to the high dust removal rate in the power industry, PM_2.5_ emissions can be ignored compared with other pollutants [52]. It can be seen that the reduction of SO_2_ emissions in the power sector would have significant health co-benefits, with the number of cases of chest discomfort decreasing between 2020 and 2050. Figure 2 shows the health effects of SO_2_ emissions under different scenarios in the power sector. By comparing different scenarios, Scenario 4 (PDC integrated scenario) had the lowest SO_2_ emissions, resulting in the lowest number of cases of chest discomfort, estimated at 72,700 in 2020 and expected to be reduced to 6700 by 2050. Scenario 1 could reduce 77,600 cases of chest discomfort between 2020 and 2050 and Scenario 2 could reduce 79,900 cases. In comparison, the reduction in SO_2_ emissions under the MPD integrated scenario had the greatest health co-benefits. Therefore, power demand control in consumption and fuel technology upgrades can significantly reduce sectoral emission levels and bring significant health co-benefits.

**Figure 3:**
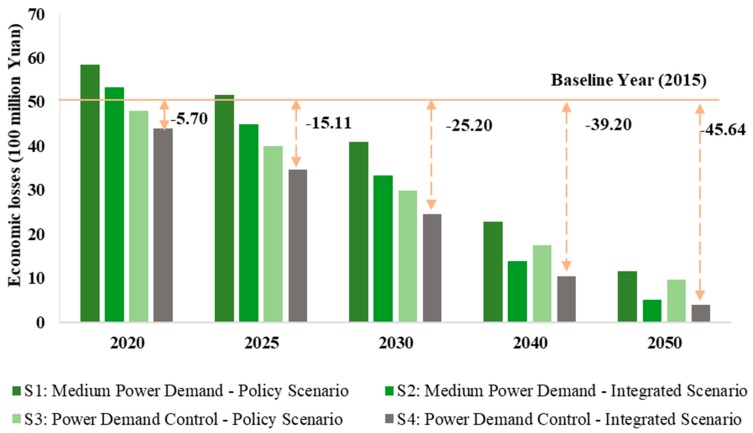
Health economic losses of SO_2_ emissions under different scenarios in the power sector.

Figure 3 shows the health economic losses of SO_2_ emissions from the power sector under different scenarios. Scenario 1 had the largest health economic losses, while Scenario 4 had the lowest. From the perspective of reducing economic losses in each scenario, Scenario 1 could reduce economic losses by 4.69 billion Yuan between 2020 and 2050. Scenario 2 could reduce losses by 4.83 billion Yuan, while Scenarios 3 and 4 could reduce losses by 3.84 billion and 4 billion Yuan, respectively. The reduction of SO_2_ emissions under the MPD integrated scenario could minimise economic losses caused by health effects. Therefore, emission reduction in the power sector can continually weaken the health effects of pollutant emissions and this co-benefit of health can offset the costs of emission reduction to some extent.

**Figure 4:**
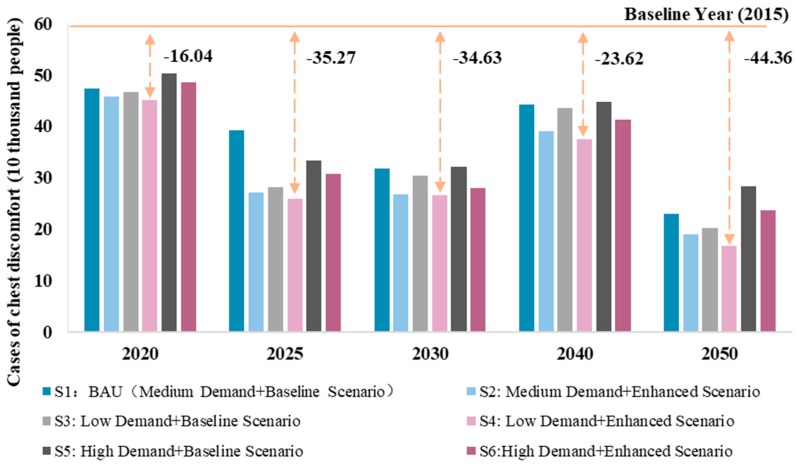
Health effects of SO_2_ emissions under different scenarios in the steel sector.

Figure 4 shows the health effects of SO_2_ emissions under different scenarios in the steel sector. It can be seen that the number of cases of chest discomfort under the 2015 baseline scenario was approximately 600,000. There should be a slight increase in 2025 and this increase could continue until after 2040. Therefore, the health co-benefits of SO_2_ emissions in the steel sector revealed a fluctuating trend, with inflection points in 2025 and 2040. Under different scenarios, Scenario 4 had the largest reduction in the number of cases of chest discomfort, while Scenario 5 had the lower. As a result, low steel demand can bring relatively greater health co-benefits, but some uncertainty remains.

**Figure 5:**
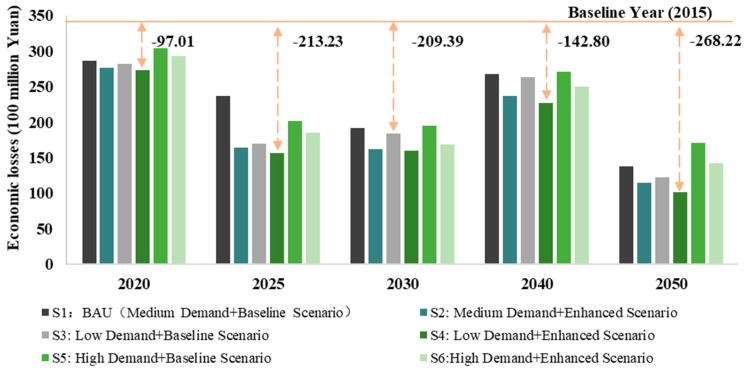
Health economic losses of SO_2_ emissions under different scenarios in the steel sector.

This study further calculated the health economic losses of SO_2_ emissions under different scenarios in the steel sector. As shown in Figure 5, economic losses had the same trend as health effects, with the same inflection points. Scenario 6 had the largest economic losses, while Scenario 3 had the lowest. Therefore, controlling steel demand can reduce the economic losses caused by the health effects of air pollution. From the perspective of reducing economic losses in each scenario, SO_2_ reduction in Scenario 4 minimised economic losses.

**Figure 6:**
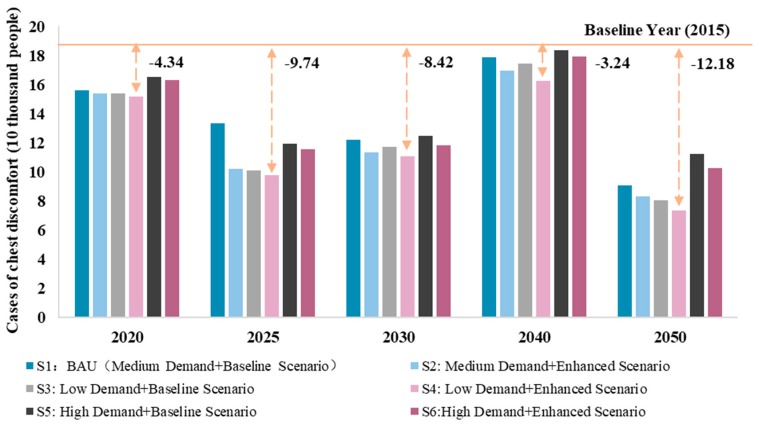
Health effects of PM_2.5_ emissions under different scenarios in the steel sector.

In addition, this study estimated the health effects of PM_2.5_ emissions in the steel sector under different scenarios. As shown in Figure 6, compared with SO_2_ emissions, PM_2.5_ emissions had a relatively small effect on health, but followed the same trend as SO_2_ emissions. The number of cases of chest discomfort under the 2015 baseline scenario was approximately 200,000. After 2015, the number of cases gradually decreased. However, there should be a slight increase in 2025 and this increase could continue until after 2040. Therefore, the reduction of PM_2.5_ emissions still had some uncertainty. Under different scenarios, Scenario 4 had the largest reduction in the number of cases of chest discomfort, while Scenario 5 had the lowest, indicating the urgency and need to reduce steel demand.

**Figure 7:**
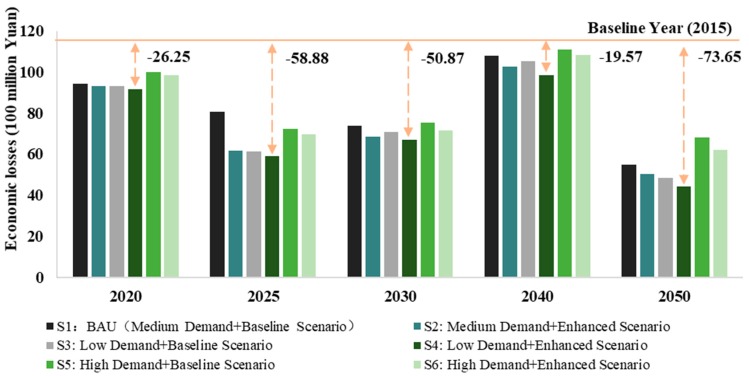
Health economic losses of PM_2.5_ emissions under different scenarios in the steel sector.

Figure 7 shows the health economic losses of PM_2.5_ emissions in the steel sector under different scenarios. In contrast to the health effects in Figure 6, Scenario 5 had the largest economic losses, while Scenario 4 had the lowest. However, it can be concluded that controlling the demand for steel can reduce the economic losses caused by the health effects of air pollution. From the perspective of reducing economic losses in each scenario, the reduction of SO_2_ emissions in Scenario 4 minimised economic losses, which was the same as SO_2_ emissions. Therefore, according to the emission reduction scenarios for the steel sector, there was still some uncertainty in terms of health co-benefits. A possible reason may be that new sources of pollution may appear in the inflection point years.

Current studies have suggested that health benefits can offset the costs of energy conservation and emission reduction policies. Some studies have shown that even a small increase in air pollutants would lead to increased health costs. Crawford-Brown et al. [37] considered that by 2020, Mexico’s mitigation policy would result in the reduction of 3000 deaths and 417,000 non-fatal diseases per year. In addition, some studies have quantified the health co-benefits of emission reduction, suggesting that the reduction of one ton of CO_2_ emissions would save between US $2 and US $380 [62] and the reduction of one ton of CH_4_ emissions would save between US $700 and US $5000 [63,64]. The results of this study are consistent with the current literature.

## 5. Conclusions

Air pollution has become one of the most serious environmental problems and one of the biggest challenges for China’s development. In particular, SO_2_ and PM_2.5_ have seriously affected human health and led to huge health costs. The results of this study show that SO_2_ and PM_2.5_ emissions in energy intensive sectors led to approximately 850,000 premature deaths, and 10 million cases of respiratory diseases and chest discomfort, resulting in health-related economic losses of 1.2 trillion Yuan, representing 1.6% of GDP. Therefore, in the face of severe environmental pollution and increasingly prominent health effects, there is a need to focus on the health effects of energy intensive sectors and to use health co-benefits to better promote energy conservation and emission reduction.

Among the 10 energy intensive sectors examined in this study, the health-related economic losses caused by the power sector and the metal processing sector accounted for 80% of all energy intensive sectors. Therefore, this study used these two industries as examples to measure the potential health co-benefits of low carbon transition scenarios. The predictions showed that power demand control in consumption and fuel technology upgrades would significantly reduce overall SO_2_ emissions from the power sector and have obvious health co-benefits. The reduction of SO_2_ emissions under the Low Demand Enhanced Scenario in the steel sector would minimise the health effects and economic losses caused by air pollution, but the health co-benefits of PM_2.5_ emission reduction were highly uncertain, particularly in the inflection point years when new sources of pollution may appear.

The Chinese government has taken active measures to combat air pollution, such as the new National Ambient Air Quality Standards, the 12th Five-Year Plan on Air Pollution Prevention and Control in Key Regions and the national Air Pollution Prevention and Control Action Plan (2013–2017). Despite these measures, government environmental policies do not pay enough attention to health issues. To analyse the characteristics and trends of the effects of haze on human health, it is necessary to establish a haze health effect monitoring network nationwide through systematic and long-term monitoring, to assess the exposure level to characteristic pollutants under haze weather, health risks and characteristics, people prone to haze-related diseases, susceptible populations and regional differences. As a result, the government can study and publish environmental policies and interventions to reduce health risks.

The results of this study show that the health effects of pollutant emissions from energy intensive sectors were significant. SO_2_ and PM_2.5_ emissions caused nearly 1 million premature deaths, leading to huge health and economic losses. Therefore, in the process of pollution control and health interventions in China, we should pay attention to the health effects of energy intensive sectors and their related health economic losses. From a technical perspective, energy intensive sectors should strengthen and promote their transformation into a cleaner approach. In the power sector, market-oriented reforms and cross-regional transmission of clean electricity will help achieve green transformation. Using smart public policy to target the production of the few energy-intensive commodities can also have a major positive impact. For the steel sector, eliminating backward production capacity, developing short-process steelmaking, energy-saving technological transformation and technological innovation can promote the green transformation. Therefore, we should focus on promoting the transformation of energy intensive sectors, mainly the power sector, by promoting the reform of the electric power system and strengthening unified planning in the power sector.

In addition to mitigating greenhouse gas emissions from energy intensive sectors, the results also revealed certain co-benefits of reducing pollutant emissions and related negative health effects. The study shows that the health co-benefits of carbon reduction in the power sector would be significant and the momentum of transformation should be maintained, promoting the low carbon transition of the power sector. In the process of energy conservation and emission reduction, for sectors with obvious health co-benefits, policy instruments should be used more flexibly, for example by encouraging clean transformation with various subsidies or rewards related to the health co-benefits, which may partly offset the costs of emission reduction.

Finally, due to the limitation of data and the measurement of ER relationship coefficients, this study estimated two pollutants and selected two sectors as cases, the health effects of many environmental pollutants, as well as emerging pollutants, should be well-studied in the future. With the continuous emphasis on China’s emission reduction issues, it is hoped that in future studies, the sources and emission reduction strategies of negative health effects in more sub-sectors, as well as the sub regional exposure response assessment could be explored.

## Figures and Tables

**Table 1:** Measures of exposure response and health effects of SO_2_ and PM_2.5_ emissions.

Health Effect	Exposure Response Cases per Million per ug/m^3^	Economic Losses (Yuan, 2016)	
Due to SO_2_		Ho and Jorgenson	Revised
Premature mortality (deaths)	1.95	370,000	394,790
Chest discomfort (cases)	10,000	6.2	6.6154
Respiratory symptoms/child (cases)	5	6.2	6.6154
Due to PM_2.5_	Exposure Response Cases per Million per ug/m^3^	Economic Losses Low (Billion Yuan)	Economic Losses High (Billion Yuan)
Total mortality (not accidental)	20.73	6.67 × 10^−3^	1.33 × 10^−2^
Mortality (respiratory diseases)	2.46	6.68 × 10^−3^	1.33 × 10^−2^
Mortality (circulatory diseases)	6.2	6.67 × 10^−3^	1.33 × 10^−2^

Notes: The dose response coefficient of Ho and Jorgenson was based on Word Bank 1997, the revised coefficient in this paper is based on price index 2016.

**Table 2:** Revised inhalation factors for primary and secondary pollutants.

Sector	Global Inhalation Factor		Secondary Particulate Matter	
	TSP	SO_2_	SO_2_/SO_4_	PM2.5
Agriculture	2.23696 × 10^−6^	4.82239 × 10^−7^	6.39613 × 10^−6^	1.13666 × 10^−5^
Coal mining and processing	1.52878 × 10^−5^	2.40557 × 10^−5^	6.39613 × 10^−6^	1.13666 × 10^−5^
Crude petroleum mining	1.52878 × 10^−5^	2.40557 × 10^−5^	6.39613 × 10^−6^	1.13666 × 10^−5^
Natural gas mining	1.52878 × 10^−5^	2.40557 × 10^−5^	6.39613 × 10^−6^	1.13666 × 10^−5^
Metal ore mining	1.52878 × 10^−5^	2.40557 × 10^−5^	6.39613 × 10^−6^	1.13666 × 10^−5^
Non-ferrous mineral mining	1.52878 × 10^−5^	2.40557 × 10^−5^	6.39613 × 10^−6^	1.13666 × 10^−5^
Building materials	1.50629 × 10^−5^	1.74236 × 10^−5^	6.39613 × 10^−6^	1.13666 × 10^−5^
Food products	1.52878 × 10^−5^	2.40557 × 10^−5^	6.39613 × 10^−6^	1.13666 × 10^−5^
Textile goods	1.52878 × 10^−5^	2.40557 × 10^−5^	6.39613 × 10^−6^	1.13666 × 10^−5^
Apparel, leather	1.52878 × 10^−5^	2.40557 × 10^−5^	6.39613 × 10^−6^	1.13666 × 10^−5^
Sawmills and furniture	1.52878 × 10^−5^	2.40557 × 10^−5^	6.39613 × 10^−6^	1.13666 × 10^−5^
Paper products	1.52878 × 10^−5^	2.40557 × 10^−5^	6.39613 × 10^−6^	1.13666 × 10^−5^
Petroleum refining	1.52878 × 10^−5^	2.40557 × 10^−5^	6.39613 × 10^−6^	1.13666 × 10^−5^
Coking	1.52878 × 10^−5^	2.40557 × 10^−5^	6.39613 × 10^−6^	1.13666 × 10^−5^
Chemical	1.42761 × 10^−5^	2.8777 × 10^−5^	6.39613 × 10^−6^	1.13666 × 10^−5^
Metal products	1.64119 × 10^−5^	2.63039 × 10^−5^	6.39613 × 10^−6^	1.13666 × 10^−5^
Machinery and equipment	1.52878 × 10^−5^	2.40557 × 10^−5^	6.39613 × 10^−6^	1.13666 × 10^−5^
Other manufacturing	1.64119 × 10^−5^	2.63039 × 10^−5^	6.39613 × 10^−6^	1.13666 × 10^−5^
Electrical products	6.27248 × 10^−6^	6.91322 × 10^−6^	6.39613 × 10^−6^	1.13666 × 10^−5^
Gas production and supply	1.52878 × 10^−5^	2.40557 × 10^−5^	6.39613 × 10^−6^	1.13666 × 10^−5^
Aquaculture	1.52878 × 10^−5^	2.40557 × 10^−5^	6.39613 × 10^−6^	1.13666 × 10^−5^
Construction	8.40827 × 10^−5^	2.40557 × 10^−5^	6.39613 × 10^−6^	1.13666 × 10^−5^
Transport & warehousing	5.46313 × 10^−5^	2.40557 × 10^−5^	6.39613 × 10^−6^	1.13666 × 10^−5^
Social services	5.60926 × 10^−5^	2.40557 × 10^−5^	6.39613 × 10^−6^	1.13666 × 10^−5^

**Table 3:** Scenario descriptions in the power sector.

Scenarios	Description
BAU	Follow the existing green policies ^a^ The share of renewable electricity generation maintains the same changing trends ^b^
MPD-Policy	When the power demand maintains a medium-speed growth, each region should first meet the policy plan between 2015 and 2020
MPD-Integrated	Follow the existing green policies The share of renewable electricity generation maintains the same changing trends Improve the energy efficiency for coal-fired power generation technologies ^c^ Promote the technologies of SC, USC, CFB, IGCC and nuclear power plants
PDC-Policy	Power demand will be controlled from the consumption side in the future When the socialist modernization is basically realized by 2035, China’s per capita electricity consumption will be 6336 kWh/person. By 2050, China’s per capita electricity consumption will reach 8323 kWh/ person and each region will first meet policy planning between 2015 and 2020 Follow the existing green policies Increase the share of renewable electricity generation
PDC-Integrated	Based on the policy scenario, the fuel consumption rate of coal-fired power generation technologies in each region will remain 2% lower every five years from 2015 Follow the existing green policies Improve the energy efficiency for coal-fired electricity generation technologies Promote the technologies of SC, USC, CFB, IGCC and nuclear power plants Increase the share of renewable electricity generation

Note: ^a^ Policies we considered are ‘The 13th Five Year Plan of Power Development’ [53] and ‘The Emissions Reduction Action Plan of the Transformation and Upgrading of Coal-fired Power’ (2014–2020) [54]. ^b^ According to ‘China Electric Power Yearbook 2011–2016′, we assumed that the shares of renewable electricity generation after 2015 maintain the same changing trends of every five years between 2010 and 2015. ^c^ According to Guo et al.’s work (2016) [55], we assumed that the energy consumption rates for all coal-fired power generation technologies decline by 2% every 5 years. BAU = business as usual. MPD = medium power demand. PDC = power demand control.

**Table 4:** Scenario descriptions in the steel sector.

Scenarios	Description
BAU (Medium Demand + Baseline Scenario)	The baseline scenario refers to a scenario based on the development of existing policies and technologies ^a^ Eliminating backward production capacities and small-scale equipment such as blast furnaces, converters, and EAFs, and increasing the proportion of large-scale and internationally advanced traditional equipment The new and rebuilt equipment will meet the planning requirements
Low Demand + Baseline Scenario	Low demand is based on the GDP growth rate and population of SSP4 Follow the existing policies and technologies
High Demand + Baseline Scenario	High demand is based on the high-speed population scenario of United Nations and the predicted GDP data Follow the existing policies and technologies
Medium Demand + Enhanced Scenario	The enhanced scenario refers to the increased use of electric arc furnaces and the acceleration of the development of energy-saving technologies On the basis of baseline scenario, more low-carbon devices will be added, such as dry-quenching, dry-cleaning, and waste heat recovery. At the same time, non-blast furnace ironmaking will be developed properly
Low Demand + Enhanced Scenario	Low demand is based on the GDP growth rate and population of SSP4 Follow the existing green policies On the basis of baseline scenario, more low-carbon devices will be added
High Demand + Enhanced Scenario	High demand is based on the high-speed population scenario of United Nations and the predicted GDP data On the basis of baseline scenario, more low-carbon devices will be added

Note: ^a^ Policies we considered are ‘The directory of national key energy-saving and low-carbon technologies promotion’ [57] and ‘China’s 13th of five-year national energy technology innovation planning’ [58].

**Table 5:** Health effects of SO_2_ and PM_2.5_ emissions in energy intensive sectors (2016).

Health Effects	Cases/Person
Due to SO_2_	
Premature mortality	46,863
Chest discomfort	23,431,695
Respiratory symptoms/child	5,857,924
Due to PM_2.5_	
Total mortality (not accidental)	800,120
Mortality (respiratory diseases)	98,415
Mortality (circulatory diseases)	30,509

**Table 6:** Economic losses of SO_2_ and PM_2.5_ emissions in energy intensive sectors (2016).

Economic Losses	100 Million Yuan
Due to SO_2_	
Premature mortality	1338.04
Chest discomfort	1121.06
Respiratory symptoms/child	280.26
Total	2739.36
Proportion of GDP	0.37%
Due to PM_2.5_	
Total mortality (not accidental)	8000.8
Mortality (respiratory diseases)	985.13
Mortality (circulatory diseases)	305.07
Total	9291
Proportion of GDP	1.25%
